# Takotsubo syndrome following mitral transcatheter edge-to-edge repair: a case report and literature review

**DOI:** 10.3389/fcvm.2025.1516080

**Published:** 2025-03-11

**Authors:** Si Pang, Haobo Huang, Yunlong Zhu, Rong Zhou, Dan Tan, Yuqing Zhang, Mingxing Wu

**Affiliations:** Department of Cardiology, Xiangtan Central Hospital (The Affiliated Hospital of Hunan University), Xiangtan, China

**Keywords:** mitral valve repair, Takotsubo syndrome, postoperative complications, mitral regurgitation, Takotsubo cardiomyopathy

## Abstract

**Background:**

Takotsubo syndrome (TTS), characterized by transient wall motion aberrations and clinical manifestations akin to acute coronary syndrome, predominantly arises from significant physical or emotional stress, often throughout the perioperative period. The prevalence and mechanisms of this condition remain inadequately elucidated, particularly in the context of transcatheter valvular disease procedures. This knowledge gap may result in under-recognition and subsequent delays in diagnosis.

**Case summary:**

A 76-year-old female was scheduled in our department for mitral transcatheter edge-to-edge repair (TEER). Despite the procedural success, multi-lead T-wave inversions and a 43% decrease in ejection fraction accompanied by new apical hypokinesis were noted postoperatively. Subsequent assessment revealed TTS. After receiving the optimal medical therapy, the patient was discharged after 10 days without experiencing acute chest pain or shortness of breath. The patient’s electrocardiogram (ECG) and function of the left ventricular function, particularly regional wall motion abnormalities, recovered on the 20th day after surgery.

**Discussion:**

The limited literature reporting TTS post-TEER that we reviewed suggests that this rare complication must be anticipated in patients exhibiting an unexpected postoperative ECG and impaired myocardial contraction.

**Conclusion:**

Researchers call for high-risk patient identification, adequate preoperative evaluation, and vigilant postoperative monitoring, and note the significance of early detection in optimizing therapeutic outcomes. Further research is imperative to further explore the management and prognosis of TTS following TEER.

## 1 Introduction

Takotsubo syndrome (TTS), also known as “Takotsubo cardiomyopathy,” “stress-induced cardiomyopathy,” or “broken heart syndrome,” was initially described in the Japanese literature in the early 1990s (1). It manifests as a transient left ventricle dysfunction, presenting with apical ballooning or mid-ventricular, basal, or focal wall motion abnormalities, and the clinical symptoms and signs usually simulate acute coronary syndromes (2, 3). This condition is frequently triggered by sudden emotional or physical stress, particularly during the perioperative period following cardiac and non-cardiac surgery (4). However, a comparable occurrence has yet to be thoroughly explained following mitral transcatheter edge-to-edge repair (TEER), which has reignited the debate regarding the publication mechanism and prognosis of TTS.

Herein, we present a distinctive instance of TTS following a successful TEER, analyze the potential etiology, and highlight early indicators of this situation. In addition, we searched PubMed with keywords related to “TEER” or “Mitral valve repair” along with “Takotsubo syndrome,” “Takotsubo cardiomyopathy,” or “left ventricular dysfunction” to conduct a brief review on TTS after the TEER procedure.

## 2 Case presentation

**Figure 1:**
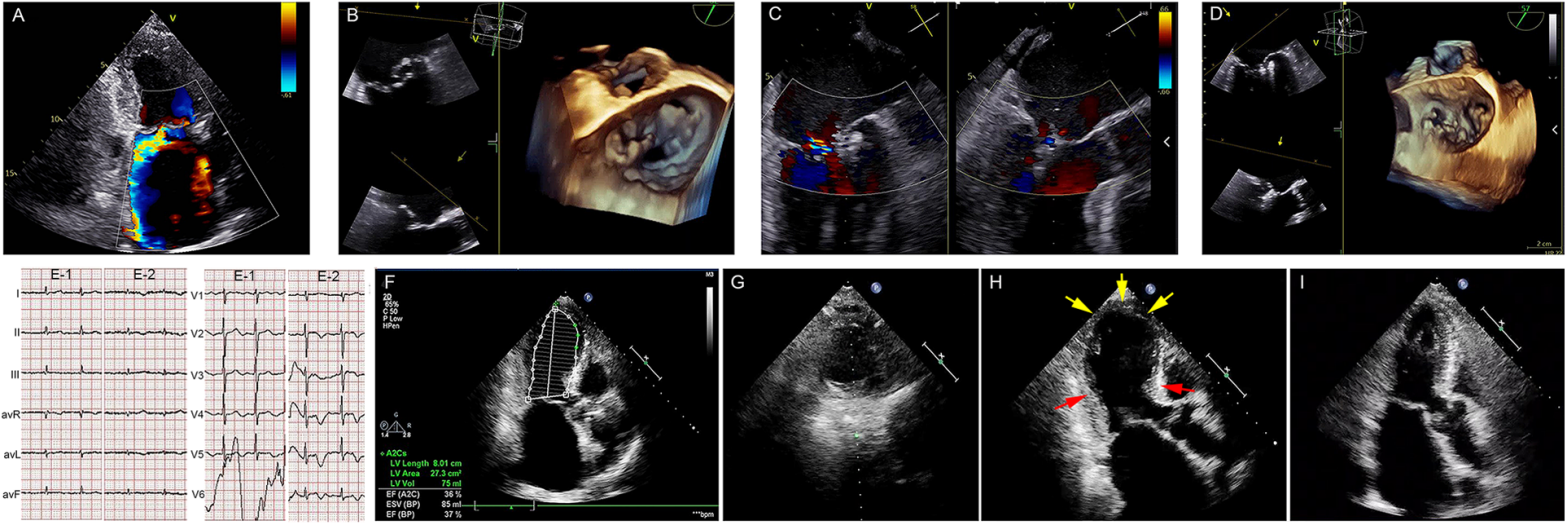
Takotsubo syndrome following mitral transcatheter edge-to-edge repair. (**A**) Preprocedural TTE. (**B**) Preprocedural 3D-TEE. (**C,D**) X-plane view with color flow and postprocedural TEE demonstrating mild MR after implantation of two clips. (**E-1**) ECG on admission and (**E-2**) postprocedural day 1. (**F–G**) Postprocedural TTE showing a significant reduction in LV function. (**H**) TTE showing apical akinesia (yellow arrowheads) and basal hyperkinesia (red arrows) on postoperative day 4. (**I**) Postoperative TTE on postoperative day 20 showing LV function and apical contraction recovery. TTE, transthoracic echocardiography; 3D-TEE, three-dimensional transesophageal echocardiograph; MR, mitral regurgitation; LV, left ventricle.

A 76-year-old woman (body mass index: 24.97 kg/m^2^) was admitted for symptomatic moderate to severe mitral regurgitation (MR). She was experiencing chest tightness and palpitations, her physical activity was mildly restricted, and she was classified as NYHA functional class II. The patient had no notable medical history and was not receiving treatment. Cardiac auscultation revealed a systolic murmur at the mitral focus assessed at 3/6. The electrocardiogram (ECG) on admission revealed an atrial fibrillation rhythm with an average ventricular rate of 90 bpm with no significant ST-T abnormalities. Transthoracic echocardiography (TTE) demonstrated a left ventricular (LV) ejection fraction of 71%, with moderate to severe MR (Figure 1A), moderate tricuspid regurgitation, and pulmonary arterial hypertension. Transesophageal echocardiography (TEE) confirmed a mitral valve area of 5.62 cm^2^, absent mitral stenosis, and a severe MR jet caused by a prolapse of the posterior mitral leaflet, measuring 16 mm in length (Figure 1B). The thyroid evaluation showed non-autoimmune subclinical hypothyroidism, characterized by elevated anti-thyroid stimulating hormone levels at 36.28 µIU/ml (NV: 0.34–5.6 µIU/ml), free T4 at 0.61 ng/dl (NV: 0.58–1.64 ng/dl), anti-thyroglobulin antibodies at 13.3 IU/ml (NV: <115 IU/ml), anti-thyroperoxidase antibodies at 10.9 IU/ml (NV: <34 IU/ml), and thyroid-stimulating receptor antibodies at 0.3 IU/ml (NV: <1.5 IU/L). The patient exhibited limited enthusiasm for surgical intervention. Consequently, we opted to discharge the patient with medication (rivaroxaban 10 mg/day, metoprolol 23.7 mg/day, thyroxine 50 μg/day, and furosemide 20 mg/temporary) and review her at a 3-month follow-up.

Over 2 months later, she presented to our department with progressively worsening dyspnea and decreased physical endurance. Her N-terminal pro-brain-type natriuretic peptide (NT-proBNP) level had increased from 1,623 pg/ml (NV: <1,800 pg/ml) to 2,928 pg/ml. TTE detected the progression of her mitral valve pathology with stable LV systolic function at 65%, and worsening MR due to a newly discovered chordae tendineae rupture (Supplementary Video 1). Subsequent coronary angiography detected no significant coronary lesions.

Given the high surgical risk and patient resistance, a TEER using the MitraClip device was carried out following a multidisciplinary heart team's deliberation (Figures 1C,D). At the beginning of the surgery, the patient's vitals were recorded as blood pressure of 100/74 mmHg, heart rate of 80 beats/min, and oxygen saturation of 98% on room air. Under general anesthesia, mild hypotension was managed with minimal doses of methoxamine, and transcatheter mitral valve repair was successfully performed using the MitraClip NT device manufactured by Abbott Vascular in Santa Clara, CA, USA (Supplementary Videos 2, 3). Continuous intraoperative monitoring was conducted using three-dimensional TEE and fluoroscopy. The MitraClip was deployed in the left atrium post-atrial septum puncture. The initial clip was placed at the A1-P1 coaptation site of the mitral valve, yielding moderate residual regurgitation. A second clip was successfully positioned at the A2-P2 coaptation site, resulting in minimal residual regurgitation and cessation of pulmonary venous reflux. The mean trans-mitral pressure gradient measured 2.5 mmHg. During the 215-min procedure, the patient's hemodynamics remained stable, without complications, and she was subsequently returned to the ward.

On postoperative day 1, the patient experienced no acute chest pain or dyspnea, and the invasive blood pressure monitoring showed fluctuations between 106-131/59-72 mmHg. The 12-lead ECG revealed significant, symmetric T-wave inversion across leads I, II, avL, and precordial leads V1–V6 (Figure 1E). A bedside cardiac ultrasound revealed no new changes. The patient received routine oral anti-heart failure medications and was moved back to the regular ward for recovery without incidents. By postoperative day 4, a follow-up TTE showed significantly reduced apical movement and a decreased left ventricular ejection fraction (LVEF) of 37%, although basal segment contraction had increased (Figures 1F–H, Supplementary Video 4). This unexpected finding prompted an immediate reevaluation, which disclosed a significant rise in NT-proBNP levels to 6,044 pg/ml and a modest rise in cardiac high-sensibility troponin T levels to 0.029 ng/ml (NV: 0.003–0.014 ng/ml).

The diagnosis of TTS was confirmed through the elimination process. The therapeutic regimen included rivaroxaban 15 mg/day, metoprolol 47.5 mg/day, sacubitril/valsartan 50 mg/day, spironolactone 20 mg/day, furosemide 20 mg/2 days, potassium chloride 500 mg/2 days, thyroxine 62.5 μg/day, and qili qiangxin capsules 3.6 g/day. The patient was discharged 10 days post-therapy, following stabilization of her LVEF and ongoing medication adherence. A 20-day follow-up confirmed her improved clinical status, as she was classified as NYHA class I and had restored ventricular kinesis showing a normalized ejection fraction (Figure 1I, Supplementary Video 4, and Supplementary Figure S1).

## 3 Discussion

Takotsubo syndrome has been described during various cardiac procedures, such as mitral valve replacement, due to factors such as surgical stress, cardiopulmonary bypass, and cardioplegic arrest (5). However, to our knowledge, this account of a TEER-related complication, which is both rare and potentially life-threatening, is unique. Following the TEER procedure, there was a notable decline in the patient’s LVEF and an occurrence of silent wall movement disorders. The diagnosis of TTS was delayed until 4 days post-operation, due to the absence of clinical signs. Fortunately, stable hemodynamics and strict adherence to prescribed medications contributed to a favorable outcome.

According to Mayo and InterTAK criteria, the clinical diagnosis of TTS is as follows: (1) transient hypokinesia, akinesia, or dyskinesia of the left ventricle, often presenting as apical ballooning or abnormalities in mid-ventricular, basal, or focal wall motions; (2) slight elevations in cardiac enzyme and biomarker levels; (3) absence of obstructive coronary disease; (4) new ECG abnormalities; and (5) excluding of other potential causes (2, 6). Our patient exhibited severe apical LV hypokinesis, T-wave inversions predominantly in the precordial leads, and a minor increase in troponin T levels, aligning with the typical TTS manifestations. Unfortunately, cardiac magnetic resonance (CMR) imaging was not performed due to concerns about the patient's ability to tolerate magnetic resonance imaging examinations and the mild nature of the symptoms. Undoubtedly, CMR can visualize the entire spectrum of functional and structural changes in those patients and provide additional value for differential diagnosis, pathophysiological insights, and complication detection (7).

The underlying pathophysiology of TTS remains intricate and is not yet fully elucidated. Previous studies have reported that the incidence of TTS more often affects postmenopausal women (>80%) with a significant burden of emotional stress and anxiety (8). In addition, hypothyroidism might also contribute to the onset of TTS. An observational study of 19,713 patients in the United States investigated the heightened connection between hypothyroidism and TTS (9). Although the nature of its exact pathophysiology is elusive, one hypothesis suggests that microvascular dysfunction may impair coronary flow reserve and myocardial perfusion (10). The patient in this report was diagnosed with subclinical hypothyroidism and treated with thyroxine and displayed persistently elevated preoperative anti-thyroid-stimulating hormone levels. Owing to the deterioration of clinical manifestations, performing surgery in patients before correcting thyroid function may have hidden dangers.

Aside from the patient's gender and age status, and the existing comorbidities mentioned above, any anesthetic or surgical intervention that significantly elevates catecholamine levels might exacerbate TTS. It is important to emphasize that the patient was administered modest doses of methoxamine to manage mild hypotension during surgery, following the induction with sevoflurane and remifentanil. It is conceivable that the development of TTS was triggered by both exogenous catecholamine administration and an endogenous catecholamine surge post-anesthetic (11). The emerging insight is that this increases the risk of experiencing TTS, especially when hypotension coincides with inadequate organ perfusion caused by peripheral vasodilation and decreased peripheral vascular resistance. A preferable approach involves pursuing fluid volume expansion.

Although it remains controversial, another possible explanation is that acute afterload mismatch following MR repair could have led to a reduction in LV systolic activity, potentially causing a low cardiac output (CO) state after the surgery. Patients undergoing mitral valve replacement surgery are known to experience a potential rise in left ventricular afterload due to the elimination of the low-resistance regurgitant flow into the left atrium (12). Early experience suggests a similar outcome with the MitraClip system appears rare. The proposed risk factors are an LVEF <40%, a dilated LV (end-diastolic dimension >6 cm, end-systolic dimension >4 cm), cardiac output <3.0 L/min or cardiac index (CI) <2.0 L/min, right ventricular dysfunction, and/or pulmonary artery systolic pressure (PASP) ≥60 mmHg (13–15). In this case, we observed the development of transient LV dysfunction shortly after TEER, which occurred in a patient with previously normal LV function, a mild dilated LV, and pulmonary hypertension, posing a unique challenge for clinical prediction.

Currently, there is a lack of consensus on the management of TTS post-TEER, with experimental methods including periprocedural volume management, dobutamine support, and mechanical assistance devices such as intra-aortic balloon pumps or Impella. Fortunately, the LV function of most of these patients recovers with no clinical sequelae. In a recent study that analyzed 317 individuals treated with successful TEER, 66.9% of patients displayed an LVEF reduction 1 month post-surgery compared to their baseline levels. This decrease was primarily due to reduced total stroke volume and widespread hypocontractility. However, surprisingly, an LVEF reduction post-TEER is associated with reduced mortality (13.3% vs. 5.7%, *P* = 0.019), and a lower readmission rate for heart failure (17.1% vs. 9.0%, *P* = 0.033), presumably indicating a more significant unloading effect of the procedure (16). This may elucidate the rapid recovery and absence of symptoms in our patient, highlighting the critical importance of the substantial reduction of valve regurgitation.

We summarized previous reports on TTS through Mitral TEER, which is presented in Table 1. The relevant information includes the epidemiological characteristics, symptoms and examination, postprocedural support, prognosis, and recovery status.

**Table 1:** Cases reported in the literature of Takotsubo syndrome after mitral transcatheter edge-to-edge repair.

Author	Age/sex	Diagnosis/history	Baseline LVEF	Number of clips	The onset of symptoms after surgery	Postprocedural LVEF	Postprocedural support	Recovery duration (days)
Tang et al. (13)	77/Male	Cardiogenic shock Dementia Multiple comorbidities	Normal	2	4 h Cardiogenic shock	15%	Emergency IABP inotropic support	13
Nomura et al. (17)	87/Male	Severe MR	NR	2	1 day Chest pain	NR	NR	6
Petro et al. (18)	92/Female	Hypertension Severe PAH	Normal	1	1 day Asymptomatic	35%–40%	Optimal medical therapy	30s
Kadosaka et al. (19)	86/Female	Heart failure Atrial fibrillation	Normal	1	4 h Nausea Chest distress	NR	NR	46
Toselli et al. (20)	88/Male	Pulmonary edema Hypertension Atrial fibrillation	Preserved	2	1 day Asymptomatic	Severe reduction	Beta-blockers Diuretics	30

LVEF, left ventricular ejection fraction; IABP, intra-aortic balloon pump; MR, mitral regurgitation; NR, not reported; PAH, pulmonary arterial hypertension.

## 4 Conclusion

This study represents the inaugural systematic brief review of TTS after the mitral TEER procedure. While the development of percutaneous mitral valve repair technology has enhanced cardiovascular symptom management, post-procedure LV dysfunction has become a challenging condition. Given the potential for unexpected clinical deterioration postoperatively, TEER should be performed at centers experienced in comprehensive preoperative preparation and diligent postoperative monitoring. Any subtle clinical symptoms and postoperative ECG changes in the patient may be the signal before adverse outcomes and necessitate a cardiac ultrasound evaluation as soon as possible. Further studies are imperative to elucidate the pathogenesis, prevention, and management of TTS following TEER.

## Data Availability

The original contributions presented in the study are included in the article/Supplementary Material, further inquiries can be directed to the corresponding author.

## Supplementary material

Supplementary Video 1 The preoperative triple chamber cardiac section image of the patient revealed significant mitral regurgitation and normal left ventricular apical contractility.

Supplementary Video 2 The patient's esophageal echocardiography immediately after surgery showed a reduction in mitral regurgitation.

Supplementary Video 3 The patient's esophageal echocardiography immediately after surgery showed a reduction in mitral regurgitation.

Supplementary Video 4 On the fourth day post-surgery, the left ventricular short axis section indicated decreased contractility in the left ventricular apex.

Supplementary Video 5 Triple chamber cardiac section view of the patient at the 20-day follow-up showed normalization of left ventricular ejection fraction and resolution of apical hypokinesia.

Supplementary Figure S1 Mild to moderate mitral regurgitation on postoperative 20-day follow-up echocardiography.
