# Supplementary figures and images for: Takotsubo syndrome following mitral transcatheter edge-to-edge repair: a case report and literature review
Source: Front Cardiovasc Med. 2025 Mar 11;12:1516080. doi: 10.3389/fcvm.2025.1516080 (PMC11934111; doi:10.3389/fcvm.2025.1516080)

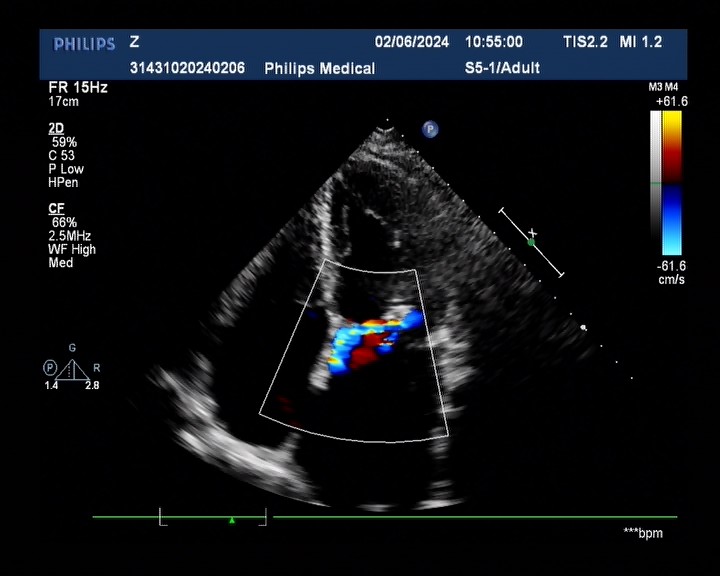

Supplement: Supplementary Figure S1 — Mild to moderate mitral regurgitation on postoperative 20-day follow-up echocardiography. [file Image1.jpeg]
